# Sickening or Healing the Heart? The Association of Ficolin-1 and Rheumatic Fever

**DOI:** 10.3389/fimmu.2018.03009

**Published:** 2018-12-18

**Authors:** Sandra Jeremias Catarino, Fabiana Antunes Andrade, Angelica Beate Winter Boldt, Luiza Guilherme, Iara Jose Messias-Reason

**Affiliations:** ^1^Molecular Immunopathology Laboratory, Department of Medical Pathology, Clinical Hospital, Federal University of Paraná, Curitiba, Brazil; ^2^Human Molecular Genetics Laboratory, Department of Genetics, Federal University of Paraná, Curitiba, Brazil; ^3^Heart Institute (InCor), School of Medicine, University of São Paulo, São Paulo, Brazil

**Keywords:** *FCN1*, ficolin-1, polymorphism, haplotype, rheumatic fever, rheumatic heart disease

## Abstract

Rheumatic fever (RF) and its subsequent progression to rheumatic heart disease (RHD) are chronic inflammatory disorders prevalent in children and adolescents in underdeveloped countries, and a contributing factor for high morbidity and mortality rates worldwide. Their primary cause is oropharynx infection by *Streptococcus pyogenes*, whose acetylated residues are recognized by ficolin-1. This is the only membrane-bound, as well as soluble activator molecule of the complement lectin pathway (LP). Although LP genetic polymorphisms are associated with RF, *FCN1* gene's role remains unknown. To understand this role, we haplotyped five *FCN1* promoter polymorphisms by sequence-specific amplification in 193 patients (138 with RHD and 55, RF only) and 193 controls, measuring ficolin-1 serum concentrations in 78 patients and 86 controls, using enzyme-linked immunosorbent assay (ELISA). Patients presented lower ficolin-1 serum levels (*p* < 0.0001), but did not differ according to cardiac commitment. Control's genotype distribution was in the Hardy-Weinberg equilibrium. Four alleles (rs2989727: *c*.−*1981A*, rs10120023: *c*.−*542A*, rs10117466: *c*.−*144A*, and rs10858293: *c.33T*), all associated with increased *FCN1* gene expression in whole blood or adipose subcutaneous tissue (*p* = 0.000001), were also associated with increased protection against the disease. They occur within the ^*^3C2 haplotype, associated with an increased protection against RF (*OR* = 0.41, *p* < 0.0001) and with higher ficolin-1 levels in patient serum (*p* = 0.03). In addition, major alleles of these same polymorphisms comprehend the most primitive ^*^1 haplotype, associated with increased susceptibility to RF (*OR* = 1.76, *p* < 0.0001). Nevertheless, instead of having a clear-cut protective role, the minor *c*.−*1981A* and *c*.−*144A* alleles were also associated with additive susceptibility to valvar stenosis and mitral insufficiency (*OR* = 3.75, *p* = 0.009 and *OR* = 3.37, *p* = 0.027, respectively). All associations were independent of age, sex or ethnicity. Thus, minor *FCN1* promoter variants may play a protective role against RF, by encouraging bacteria elimination as well as increasing gene expression and protein levels. On the other hand, they may also predispose the patients to RHD symptoms, by probably contributing to chronic inflammation and tissue injury, thus emphasizing the dual importance of ficolin-1 in both conditions.

## Introduction

Rheumatic fever (RF) is an immune-mediated disease occurring in genetically susceptible individuals, as a sequelae of group A (GAS) *Streptococcus pyogenes* pharyngitis. It affects predominantly children and adolescents in low-income and developing countries, where it remains a considerable public health problem (1, 2).

Five major manifestations reflect target tissue involvement in RF, including synovium (inflammatory arthritis), heart valves (endocarditis), brain (Sydenham's chorea), skin (erythema marginatum), and subcutaneous tissue (nodules). Repeated or severe RF episodes can result in permanent damage to the heart valves, leading to rheumatic heart disease (RHD), the most common acquired cardiovascular disease in young adults (3, 4). Rheumatic heart disease (RHD) is associated with high morbidity and mortality, causing 9 million disability-adjusted life years lost, 33 million cases (5) and 275,000 deaths each year (6). This multifactorial disorder involves multiple genetic and environmental factors, not yet fully elucidated. Well-designed case-control studies strive to unravel the genetic susceptibility to this disease, given that the only genome-wide association study done with RHD has rendered no relevant results (7).

Autoimmunity is known to play a role in the pathogenesis of RF and RHD, with tissue damage being mediated by autoantibodies resulting from molecular mimicry between GAS and heart tissue proteins. It has been shown that GAS molecules such as N-acetyl-β-D-glucosamine (GlcNAc) and M protein display cross reactivity with valve and myocellular contractile proteins of the host. GlcNAc is the immunodominant cell wall antigen of GAS, recognized by ficolins, molecules that comprise important pattern-recognition receptors (PRRs) of the complement (2, 5, 8, 9).

The complement system plays an important role, both in the defense against GAS infection, as well as in the development of autoimmunity in RHD. This system promptly responds to any pathogen, bridging innate and adaptive immune responses (10–14). Ficolins initiate one of the three complement activation pathways, known as the lectin pathway, along with collectins, as mannose-binding lectin (MBL). They occur in oligomeric structures of a basic homotrimer, where each chain is formed by a collagenous strand and a C-terminal fibrinogen-like recognition domain. The oligomers are complexed with homodimers of serine proteases (MASP-1 or MASP-2). Ficolin oligomers bind specific patterns of acetylated residues on the surface of pathogens or altered cells (8, 15). MASP-1 then autoactivates, transactivating MASP-2 leading to subsequent cleavage of downstream complement components [reviewed by Boldt et al. (16)]. The lectin pathway, along with the classical and alternative pathways, converge at the cleavage of C3 and C5, with subsequent C3b opsonization and pathogen phagocytosis or its destruction by membrane attack complex (MAC) pores on the cell membrane (17).

Three human ficolins have been described: ficolin-1 (M-ficolin), ficolin-2 (L-ficolin), and ficolin-3 (H-ficolin, Hakata antigen) (15). Unlike any other PRRs, ficolin-1 is expressed by myeloid cells, being found in monocytes, neutrophils and macrophages of the lung and spleen (8). It is also the only one that occurs in both soluble form (0.05–1.0 μg/mL in serum) and on the cell membrane (18–20). Apart from other acetylated residues, ficolin-1 has the unique ability to bind sialic acid to capsular polysaccharides of pathogens including *Streptococcus agalactiae*, as well as to the surface of immune cells (21, 22).

Ficolin-1 is encoded by the *FCN1* gene on chromosome 9q34 and contains nine exons. Among the several SNPs described for the *FCN1* gene, at least eight are associated with Ficolin-1 levels, four of them are located in the promoter and one in the first exon (23). Polymorphisms of collectin and ficolin genes have been repeatedly associated with infectious and autoimmune diseases (23–28). *FCN1* polymorphisms were associated with increased fatal outcome in patients with systemic inflammation (29), susceptibility to rheumatoid arthritis (28) and leprosy (26). Gene polymorphisms of the lectin pathway have already been associated with RF in case-control association studies with MBL (30–32), Ficolin-2 (33), and MASP-2 (34). Within this context, we are the first to investigate a membrane-bound molecule of the lectin pathway, able to activate the complement. More specifically, we evaluated the association of *FCN1* polymorphisms and haplotypes, as well as Ficolin-1 serum levels, with the susceptibility to RF and RHD.

## Materials and Methods

### Subjects and Samples

This study was approved by the local medical ethics committee (CEP/HC 2658.265/2011-11). All patients and control subjects provided written informed consent in accordance with the *Declaration of Helsinki*. We investigated a total of 193 patients with a history of RF, all of them with ASO (anti streptolysin O) titers higher than 250 units, characterizing a precedent streptococcal infection and diagnosed according to Jones' modified criteria; 55 (28.5%) males and 138 (71.5%) females; with a mean age of 37 years (range = 7–76 years). Among them, 138 had RHD, confirmed by the transthoracic echocardiogram showing rheumatic involvement of the mitral or aortic valves (Table 1), and 55 did not present RHD, but had RF history and were designated as “rheumatic fever only” (RFo) patients. None of the patients presented other inflammatory disease, neoplasia, infective endocarditis, or other infections at the time their blood was collected. Values of high-sensitivity C-reactive protein (hs-CRP) levels, C3 levels and C4 levels, previously published by our group, are shown in Table 1 (30). The control group included 193 blood donors from The Clinical Hospital of the University Federal of Paraná, with a mean age of 37 years (range = 18–64 years), 68 (35%) males, and 125 (65%) females.

**Table 1:** Clinical characteristics of patients.

**Total patients**	**193**
Males	55 (25.5%)
Females	138 (71.5%)
Mean age	37 years
Rheumatic fever only (RFo)	55 (28.5%)
Rheumatic heart disease (RHD)	138 (71.5%)
Mitral stenosis	83 (60.1%)
Aortic stenosis	25 (18.1%)
Tricuspid stenosis	4 (2.9%)
Mitral insufficiency	67 (48.5%)
Aortic insufficiency	41 (29.7%)
Tricuspid insufficiency	31 (22.5%)
hs-CRP level (mg/dL)	0.83* (0.03–26.10)
C3 level^**#**^ (mg/dL)	109.2* (50.3–198)
C4 level^**#**^ (mg/dL)	22.7* (6.82–45.4)

hs-CRP, high-sensitivity C-reactive protein. Normal reference values for hs-CRP: < 0.1 mg/dL, for C3: 82-160 mg/dL, for C4: 12-36 mg/dL.

* mean;

# *Data already published by Schafranski et al. (30)*.

### *FCN1* Genotyping

DNA extraction from peripheral blood was performed using The QIAamp DNA Blood Mini Kit, QIAGEN (Hilden, Germany), following the manufacturer's instructions. The genotype method was adapted from a previously described multiplex PCR-SSP (sequence-specific amplification) method (30). Five *FCN1* single nucleotide polymorphisms (SNPs) were genotyped: rs2989727 SNP (*c*.–*1981G* > *A*) in the distal *FCN1* promoter, rs10120023 (*c*.–*542G* > *A*), rs17039495 (*c*.–*399G* > *A*), rs10117466 (c.–*144C* > *A*), and rs10858293 (*c*.+*33T* > *G*) SNPs in the proximal *FCN1* promoter, with primers listed in Table S1.

*FCN1*_Prom-1981Af or *FCN1*_Prom-1981Gf were conjugated with the *FCN1*_Prom_r reverse primer to generate a fragment of 729 bp. PCR conditions were as follows: 0.2 μM of SSP primers, 1X Coral Load PCR buffer (Qiagen, Hilden, Germany), 1.6 mM MgCl_2_ (Qiagen, Hilden, Germany), 0.5% glycerol, 0.2 mM deoxyribonucleoside triphosphate (dNTP) (Invitrogen, São Paulo, Brazil), 0.03 U/uL of Taq polymerase (Invitrogen, São Paulo, Brazil), 0.1 μg/mL DNA, ultrapure water for 15 μL. The amplification protocol starts with a 5 min denaturation step at 94°C, followed by 10 cycles of 20 s at 94°C, 30 s at 60°C, and 30 s at 72°C; 10 cycles of 20 s at 94°C, 30 s at 56°C, and 30 s at 72°C; 10 cycles of 20 s at 94°C, 30 s at 52°C, and 30 s at 72°C, concluding with 5 min at 72°C in the final DNA extension step.

*FCN1*_Prom-542Af or *FCN1*_Prom-542Gf were conjugated with the *FCN1*_Prom-144Ar or *FCN1*_Prom-144Cr to generate a fragment of 434 bp. PCR conditions differed from those previously mentioned as follows: 0.6 μM of SSP primers, 1.7 mM MgCl_2_, and 1.5% glycerol. The amplification protocol differed from the previous one only by the annealing primer temperatures, which were 57°C in the first 10, 55°C in the next 10, and 53°C in the last 10 cycles.

*FCN1*_Prom-399Af or *FCN1*_Prom-399Gf were conjugated with the *FCN1*_Prom+33Gr or *FCN1*_Prom+33Tr to generate a fragment of 470 bp. PCR conditions differed from those previously noticed, as follows: 0.4 μM of SSP primers, 1.75 mM MgCl_2_, 0.5% glycerol. The amplification protocol differed from the first one only by the annealing primer temperatures, which were 58°C in the first 10, 56°C in the next 10 and 54°C in the final 15 cycles.

Interpretation was based on the electrophoretic pattern of the amplified fragments, on agarose gels 1.5% stained with Sybrsafe (Invitrogen, São Paulo, Brazil). This bispecific PCR-SSP approach allows the identification of 8 haplotypes: ^*^1 (GGGCG), ^*^3A (AGGCG), ^*^3A.3C2.A (AGGCT), ^*^3A.3C2.B (AGACT), ^*^3B2 (AAGCG), ^*^3C1 (AAGCT), ^*^3C2 (AAGAT), and ^*^3C2.3A (AGGCG), as previously described (26).

### Ficolin-1 Measurement

Ficolin-1 serum concentrations were measured in 78 patients and 86 controls using enzyme-linked immunosorbent assay (ELISA) SEA786Hu Cloud-Clone Corp. (Texas, USA).

### Statistics

Allele, haplotype and genotype frequencies were obtained by direct counting (the phase between distantly situated SNPs could be deduced due to the strong LD between the variants, and were verified with the Expectation-Maximization algorithm implemented in the PLINK software. Exact tests of Guo and Thompson for testing the hypothesis of Hardy-Weinberg equilibrium were accomplished using ARLEQUIN v.3.5.2.2 (http://cmpg.unibe.ch/software/arlequin35/). The investigated polymorphisms were evaluated for regulatory effects on gene expression, using information from The Genotype-Tissue Expression (GTEx) Project. Associations with alleles were tested by the Exact Fisher test using the SISA quantitative skill tables and including genotypes and haplotypes, by multivariate binary logistic regression with the software package STATA v. 9.2. Correction for associated demographic factors (sex, age) and clinical factors were applied in the reduced logistic regression model where sample size was adequate, and *p*-values ≤5% were considered significant. Distributions of ficolin-1 levels were tested for normality with the Shapiro-Wilk test. Since ficolin-1 levels presented a non-normal distribution, the two-tailed Mann-Whitney test was used to compare ficolin-1 levels between groups (GraphPad Prism v.7.03).

## Results

### *FCN1* Alleles and Haplotypes Association With RF

**Figure 1:**
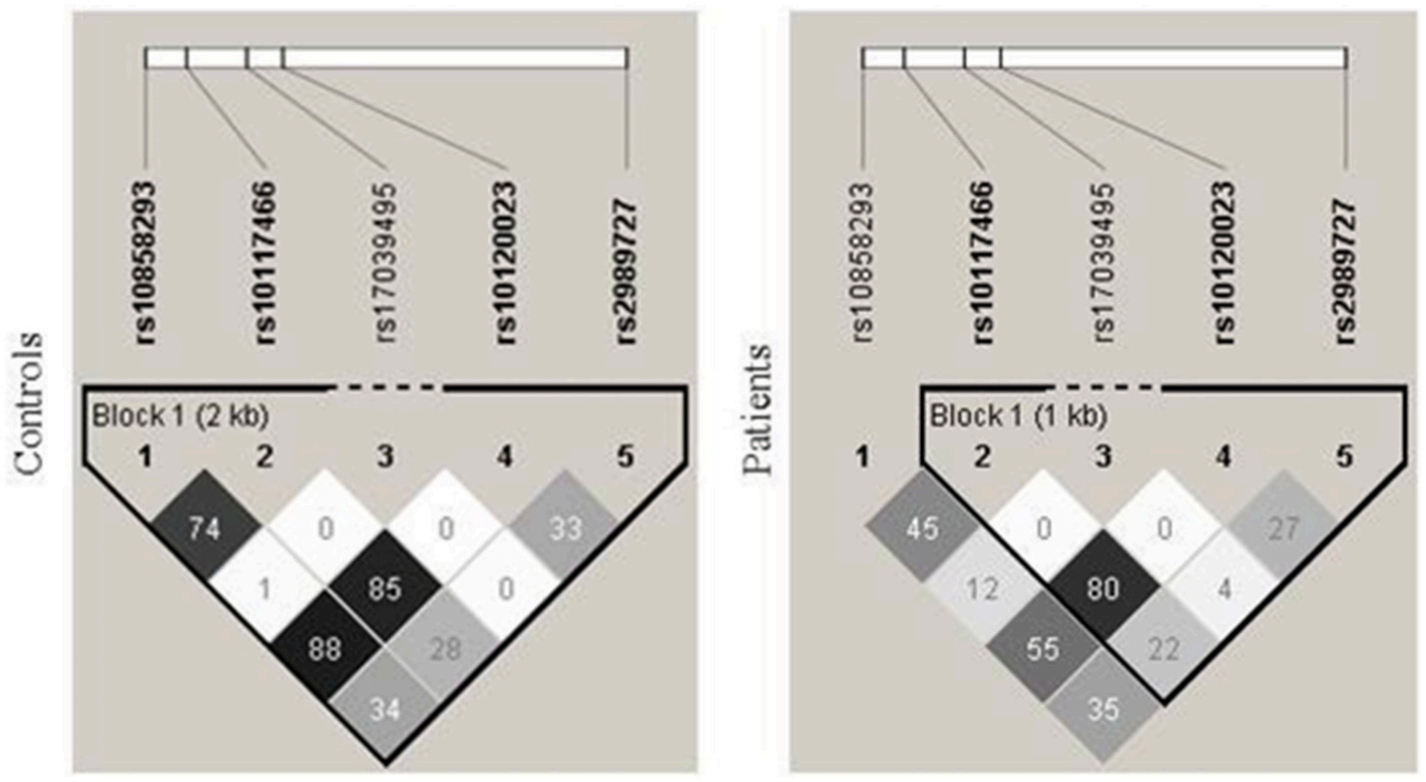
Linkage disequilibrium between *FCN1* single nucleotide polymorphisms. There was strong linkage disequilibrium (LD) between the SNPs rs10120023 (−542G > A) and rs10117466 (−144C > A), as indicated by the correlation coefficient values (r^2^). An evidence of recombination was found between the SNP rs10858293 (+33T > G) in exon 1 and the promoter variants in patients.

Genotype distribution was in equilibrium with the Hardy-Weinberg model in both controls and patients, except for SNPs rs10120023 (*c*.−*542G* > *A*) and rs10117466 (*c*.−*144C* > *A*) in patients (*p* = 0.017 and *p* = 0.024, respectively). A total of eight haplotypes were found in patients and seven, in controls. The phase between the variants in the proximal promoter was determined with bispecific PCR-SSP and the phase of these variants with the SNP *c*.−*1981G* > *A* could be deduced due to a strong linkage disequilibrium (LD). Evidence for recombination was found between the SNP rs10858293 (*c.33G* > *T*) in exon 1 and promoter variants in patients (Figure 1).

The *c*.−*1981A, c*.−*542A, c*.−*144A*, and *c.33T* alleles were associated with an increased level of protection against RF, presenting an additive effect with homozygotes protecting more than heterozygotes. All four are also known to be associated with higher *FCN1* expression in adipose subcutaneous tissue. Three of them present the same effect in peripheral blood cells (GTex portal) (35). All the protective alleles occur within the ^*^3C2 haplotype (*AAGAT*). As expected, this haplotype was also associated with increased protection against RF under the additive model (*OR* = 0.41, *p* < 0.0001). On the other hand, the common alleles of these SNPs compose the phylogenetically ancestral ^*^1 (*GGGCG*) haplotype, which was associated with increased susceptibility to RF (*OR* = 1.76, *p* < 0.0001, Table 2).

**Table 2:** *FCN1* allele, genotype and haplotype frequencies and associations with RF.

**SNP**	**Controls *N* = 193 (%)**	**Patients *N* = 193 (%)**	**RFo patients *N* = 55 (%)**	**RHD patients *N* = 138 (%)**	**Model**	**Patients vs. controls OR [95% CI]**	***P* value (*Q* = 0.00185)**	**mRNA expression NES (GTex)**
**rs2989727 (*****c.−1981G > A*****)**	230 (59.6)	177 (45.9)	53 (48.2)	124 (44.9)	*A* x G ^*^	**0.57 [0.43–0.76]**	**0.0002**	–
*G/G*	33 (17.1)	53 (27.5)	15 (27.3)	38 (27.5)	*A/A* x _	**0.42 [0.26–0.66]**	**<0.0001**	–
*G/A*	90 (46.6)	103 (53.4)	27 (49.1)	76 (55.1)	*A/_ x G/G*	0.54 [0.33-0.89]	0.014 (ns)	–
*A/A*	70 (36.3)	37 (19.1)	13 (23.6)	24 (17.4)	*A/A > A/G > G/G*	**0.57 [0.42–0.76]**	**<0.0001**	0.32 (*P* = 5.5e−10) in AS
**rs10120023 (*****c.−542G > A*****)**	127 (32.9)	73 (18.9)	27 (24.5)	46 (16.7)	*A* x G ^*^	**0.48 [0.34–0.66]**	**<0.0001**	–
*G/G*	84 (43.5)	132 (68.4)	33 (60.0)	99 (71.7)	*A/A* x __	–	ns	–
*G/A*	91 (47.2)	49 (25.4)	17 (30.1)	32 (23.2)	*A/_ x G/G*	**0.36 [0.24–0.55]**	**<0.0001**	–
*A/A*	18 (9.3)	12 (6.2)	5 (9.9)	7 (5.1)	*A/A > A/G > G/G*	**0.49 [0.35–0.68]**	**<0.0001**	0.21 (*P* = 7.4e−8) in WB 0.24 (*P* = 0.000001) in AS
**rs17039495 (*****c.−399G > A*****)**	3 (0.8)	14 (3.6)	3 (2.7)	11 (4.0)	*A* x G ^*^	4.8 [1.37–16.86]	0.012 (ns)	–
*G/G*	190 (98.4)	179 (92.7)	52 (94.5)	127 (92.0)	*A/_ x G/G*	4.7 [1.3–16.7]	0.017 (ns)	–
*G/A*	3 (1.6)	14 (7.3)	3 (5.5)	11 (8.0)	–	–	–	–
**rs10117466 (*****c.−144C > A*****)**	114 (29.5)	61 (15.8)	23 (20.9)	38 (13.8)	*A* x C ^*^	**0.45 [0.32–0.64]**	**0.000007**	–
*C/C*	92 (47.7)	141 (73.0)	36 (65.4)	105 (76.1)	*A/A* x __	–	ns	–
*C/A*	88 (45.6)	43 (22.3)	15 (27.3)	28 (20.3)	*A/_ x C/C*	**0.36 [0.21–0.60]**	**<0.0001**	–
*A/A*	13 (6.7)	9 (4.7)	4 (7.3)	5 (3.6)	*A/A > A/C > C/C*	**0.53 [0.36–0.78]**	**0.001**	0.21 (*P* = 1.1e−7) in WB 0.24 (*P* = 0.000012) in AS
**rs10858293 (*****c.33G > T*****)**	131 (33.9)	90 (23.3)	29 (26.4)	61 (22.1)	*T* x G ^*^	0.59 [0.43–0.81]	**0.0014**	–
*G/G*	83 (43.0)	114 (59.1)	30 (54.5)	84 (60.8)	*T/T* x __	0.49 [0.23–1.04]	0.064 (ns)	–
*G/T*	89 (46.1)	68 (35.2)	21 (38.2)	47 (34.1)	*T/_ x G/G*	0.52 [0.35–0.78]	0.002 (ns)	–
*T/T*	21 (10.9)	11 (6.7)	4 (7.3)	7 (5.1)	*T/T > T/G > G/G*	**0.59 [0.43–0.81]**	**0.001**	0.2 (*P* = 6.2e−7) in WB 0.26 (*P* = 0.0000011) in AS
**HAPLOTYPES**								
^*^1 (*GGGCG*)	156 (40.4)	209 (54.1)	57 (51.9)	152 (55.1)	Haplotype	**1.74 (1.31–2.32)**	**0.0002**	–
					Recessive	1.8 [1.13–3.02]	0.014 (ns)	–
					Dominant	**2.39 [1.5–3.8]**	**<0.0001**	–
					Additive	**1.76 [1.31–2.37]**	**<0.0001**	–
^*^3A (*AGGCG*)	96 (24.9)	79 (20.5)	20 (18.2)	59 (21.4)	Any	–	ns	–
^*^3C2 (*AAGAT*)	111 (28.8)	55 (14.2)	20 (18.2)	35 (12.7)	Haplotype	**0.41 [0.29–0.59]**	**<0.0001**	–
					Recessive	0.37 [0.13–1.06]	0.063 (ns)	–
					Dominant	**0.34 [0.22–0.53]**	**<0.0001**	–
					Additive	**0.41 [0.28–0.59]**	**<0.0001**	–
^*^3A.3C2.B (*AGACT*)	3 (0.8)	14 (3.6)	3 (2.7)	11 (4.0)	Haplotype	4.81 (1.37–16.86)	0.0118 (ns)	–
					Dominant	4.701 [1.32–16.69]	0.017 (ns)	–
^*^3C1 (*AAGCT*)	13 (3.4)	10 (2.6)	3 (2.7)	7 (2.5)	Any	–	Ns	–
^*^3A.3C2.A (*AGGCT*)	4 (1.0)	11 (2.8)	3 (2.7)	8 (2.9)	Dominant	2.76 [0.86–8.86]	0.087	–
^*^3C2.3A (*AAGAG*)	3 (0.8)	6 (1.6)	3 (2.7)	3 (1.1)	Any	–	Ns	–
^*^3B2 (*AAGCG*)	0	2 (0.5)	1 (0.9)	1 (0.3)	Any	–	ns	–

NES, Normalized effect size; AS, adipose subcutaneous tissue; WB, whole blood; ns, not significant Bonferroni corrected significance level q^*^ = 0.001851852.

*The values shown in bold correspond to significant values*.

In contrast, the *c*.−*1981A* allele was associated with increased susceptibility to valvar stenosis (5/19 or 26.3% vs. 6/89 or 6.7% *A/A* homozygotes and 13/19 or 68.4% vs. 39/89 or 43.8% *A/G* heterozygotes in patients with moderate to severe, vs. light or no valvar stenosis, respectively: *OR* = 3.75 [95%CI = 1.39–10.15], *p* = 0.009). Similarly, the *c*.−*144A* allele presented an increased susceptibility effect for mitral insufficiency (2/12 or 16.7% vs. 1/75 or 1.3% *A/A* homozygotes and 1/12 or 8.3% vs. 4/75 or 5.3% *A/C* heterozygotes in patients with moderate to severe vs. light or no mitral insufficiency, respectively: *OR* = 3.37 [95%CI = 1.15–9.92], *p* = 0.027). Although no other allele or haplotype were associated either with RHD or other clinical manifestation, this may be due to the relatively small sample size of RHD patients in our setting.

### Ficolin-1 Levels

**Figure 2:**
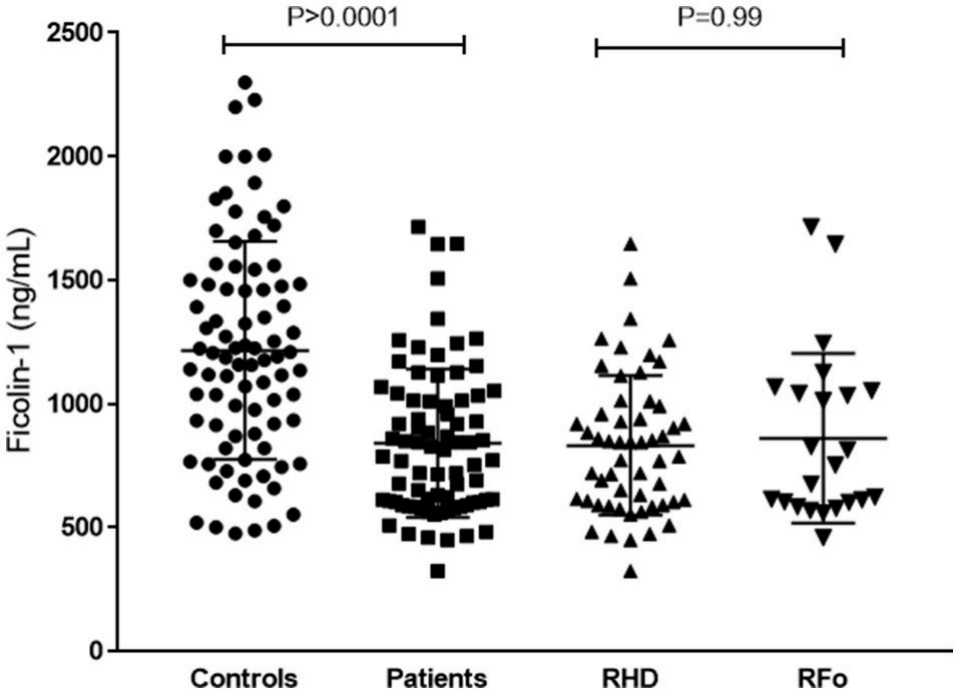
Ficolin-1 serum levels in controls and RF patients. RHD, Rheumatic Heart Disease; RFo, Rheumatic Fever only (not present RHD).

Ficolin-1 serum levels were lower in patients (median: 800.5 ng/mL [324.6–1,715 ng/mL]), compared to controls (1,208 ng/mL [488–2,852 ng/mL, *p* < 0.0001), but did not differ between RHD and RFo patient groups (Figure 2).

**Figure 3:**
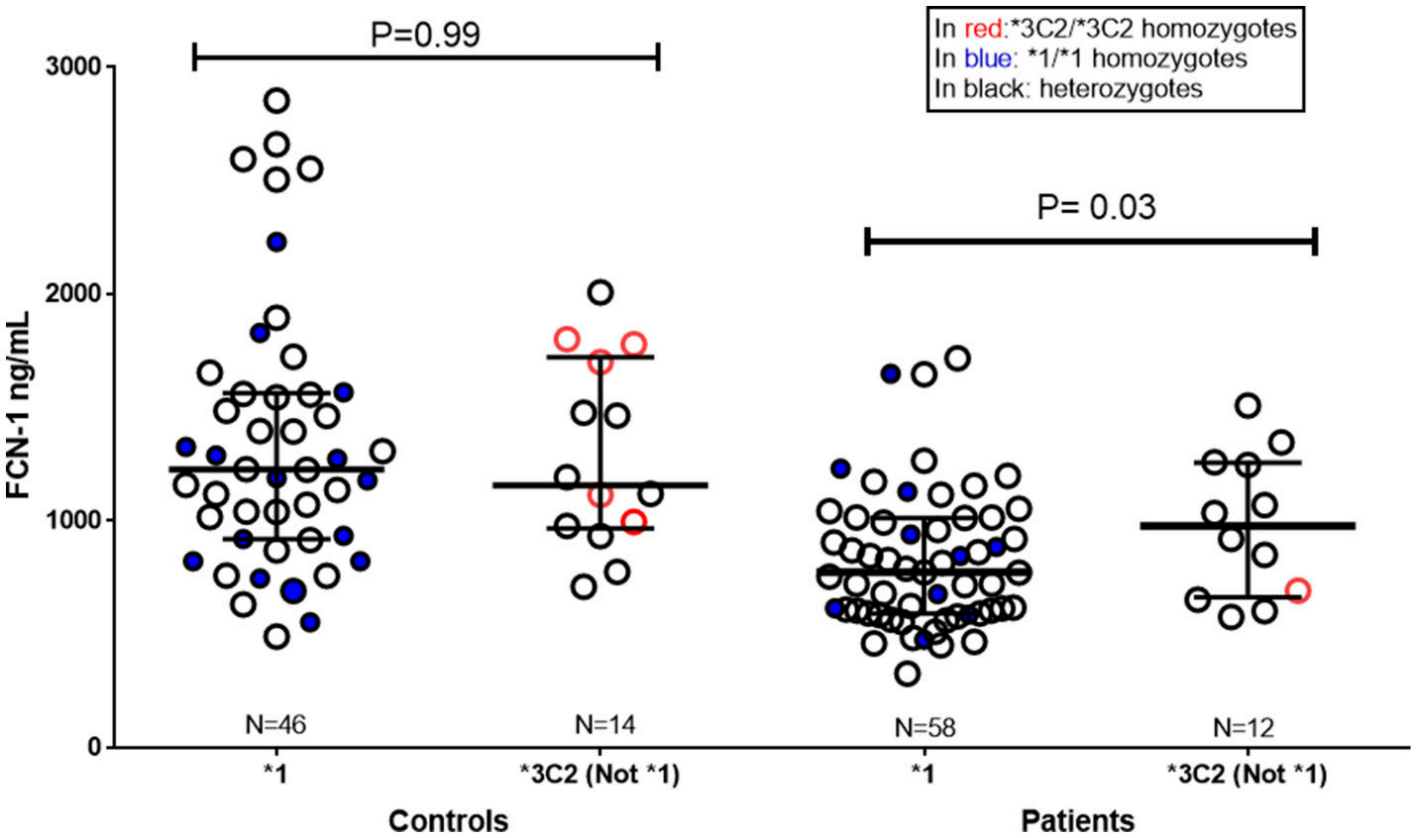
Ficolin-1 serum levels in controls and RF patients, according to *FCN1* genotype. ^*^1: carriers of the ^*^1 “risk” haplotype (*GGGCG*). Not ^*^1/^*^3C2: carriers of ^*^3C2 “protective” haplotype (*AAGAT*), but not of the ^*^1 “risk” haplotype. In red: ^*^3C2/^*^3C2 homozygotes. In blue: ^*^1/^*^1 homozygotes. Subjects carrying the other FCN1 haplotypes are not represented in the figure (Controls *N* = 26, Patients *N* = 8).

Patients with the ^*^1 (*GGGCG*) “susceptibility” haplotype presented lower ficolin-1 levels, than those with the ^*^3C2 (*AAGAT*) “protective” haplotype (*p* = 0.03, medians 770.8 and 975.9 ng/mL, respectively) (Figure 3).

## Discussion

The complement system plays an important role both in the defense against GAS infection, as well as in the development of RHD (28, 36, 37). Among studies focusing on complement genetic polymorphisms (30–34), this is the first considering the role of a complement membrane-bound molecule of the lectin pathway in the development of RF and RHD. Our results indicate that *FCN1* polymorphisms may play a dual role in the physiopathology of RF. On one hand, they increase resistance to GAS infection and on the other hand, predispose the patient to RHD symptoms, once the infection is established.

**Figure 4:**
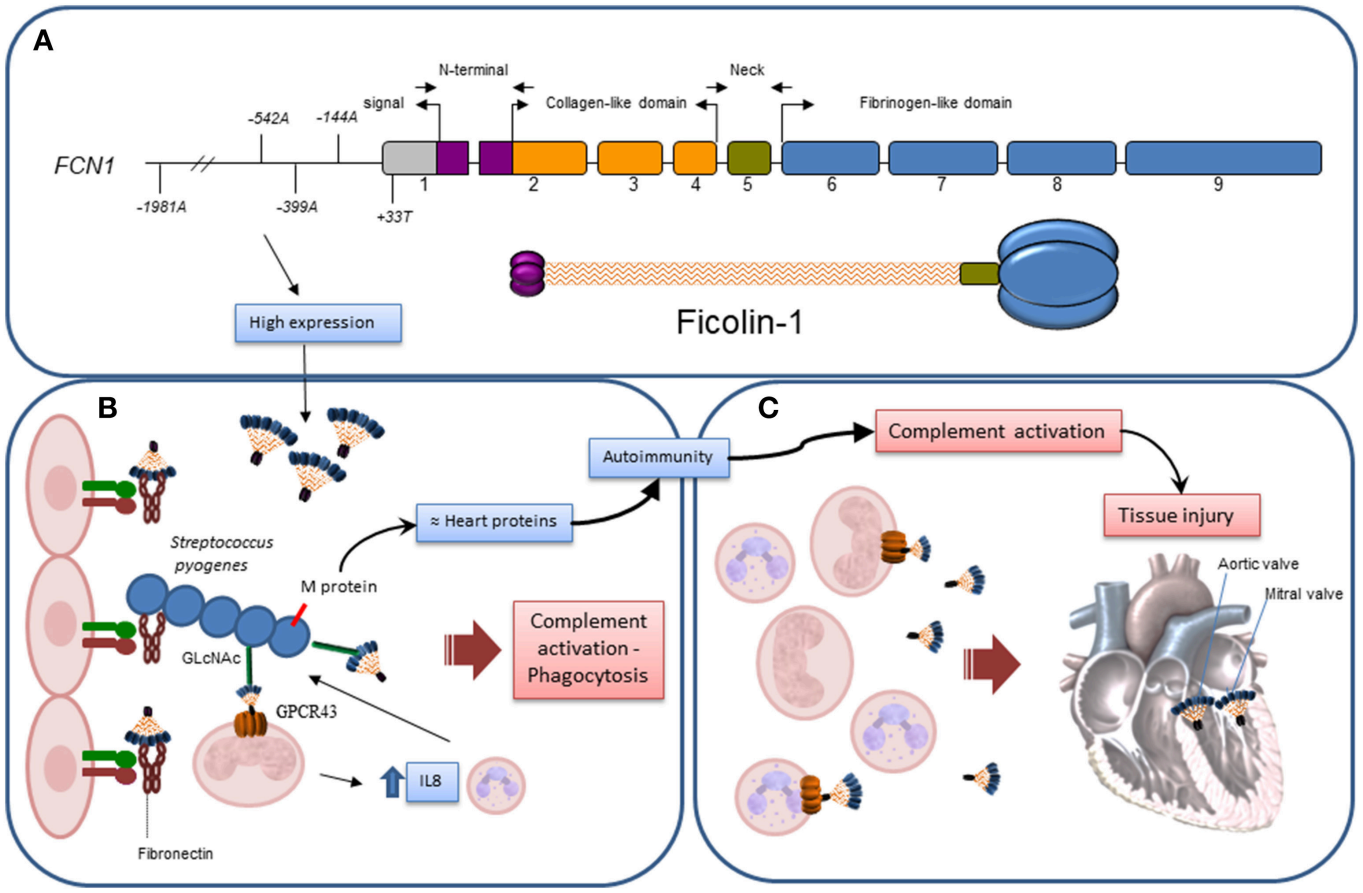
Hypothetical actions of Ficolin-1 in RF/RHD. **(A)** Exon structure and correspondence to domains in the structural trimeric ficolin-1 subunit: gray – untranslated region, purple – N-terminal trimerization domain, orange – collagenous stalk, green – neck region, blue – fibrinogen C-domain. Approximate location of the investigated polymorphisms is given according to number of base pairs, counting from the first transcribed nucleotide. **(B)** Higher ficolin-1 levels, associated with certain *FCN1* alleles in the promoter region, improved resistance against GAS infection, slowing down the process by which autoantibodies are produced due to exposure of neoepitopes in damaged tissue and structural homology of GAS's M protein with myosin/tropomyosin. This may occur through competition for fibronectin binding in the extracellular matrix; recognition of GlcNAc in GAS carbohydrate A by phagocyte membrane-bound ficolin-1 and consequent bacteria internalization; complement activation, despite GAS's abilities to cleave C5a, as well as C2-C9; ficolin-1/GPCR43 activation on monocytes, transducing signals through NFkB and activating interleukin-8 (IL8) gene expression, leading to chemoattraction of more phagocytes to the infection site. **(C)** After established infection, structures on damaged/altered cells recognized by membrane-bound or soluble ficolin-1 mediate complement activation and increases tissue injury, which may become irreversible in the cardiac valves.

A protective role for *FCN1* promoter variants against RF has been observed (Figure 4). Among the investigated *FCN1* alleles, those four occurring within the ^*^3C2 haplotype that were associated with RF protection and ficolin-1 levels have also been associated with higher *FCN1* gene expression (GTEx Portal) and ficolin-1 serum levels in other studies (23, 26, 29). Indeed, it has been suggested that the minor alleles −*542A* and −*144A* may facilitate the binding of transcription factors, causing amplified gene expression (29). Thus, it is conceivable that higher *FCN1* gene and protein expression could increase resistance against GAS infection due to ficolin-1 anti-bacterial properties. In fact ficolin-1 is able to bind sialic acid on Group B *Streptococcus* bacteria (20, 38) as well as GAS's carbohydrate A (GlcNAc) which is a preferable ficolin ligand. GAS recognition by ficolins may culminate in complement activation, despite the described complement evasion mechanisms of the bacteria, e.g., through C5a, as well as C2–C9 cleavage (39). Moreover, it is known that GAS infection occurs through fibronectin binding in the extracellular matrix (39). Fibronectin is also a ligand for ficolin-1 (40), thus competition for fibronectin binding sites may occur. Additionally, ficolin-1 anchors to GPCR43, a G-protein coupled receptor on monocytes. Ficolin-1/GPCR43 activation results in signal transduction through NFkB and interleukin-8 (IL8) gene expression. IL8 is a chemokine that attracts phagocytes to the infection site, enhancing bacterial elimination (20). Taken together, these events corroborate the eventual protective effect of ficolin-1 in the development of RF.

On the other hand, these *FCN1* variants associated with high ficolin-1 expression may contribute to excessive complement activation, leading to chronic inflammation and tissue injury, thereby predisposing the patient to RHD symptoms such as valvar stenosis and mitral insufficiency in the advanced phase of the disease. In addition, exposure to neoepitopes on bacteria-damaged valvar tissue would not only induce autoantibody production, but also promote complement activation with MAC deposition, thereby increasing tissue injury. C9, the last MAC complement, was indeed exclusively found in RHD patients with mitral stenosis, compared to control subjects (13). Thus, in the advanced phase of the disease, high ficolin-1 levels would probably not be beneficial. Even though ficolin-1 levels did not differ between RHD and RFo patients, it is possible that the quantification of ficolin-1 in serum does not reflect the levels of membrane-bound ficolin-1, and that the valvar damage could be instead related to this last ficolin-1 form. Keeping this balance—pathogen elimination vs. host preservation, has proven to be very difficult in circumstances where ineffective pathogen elimination induces chronic persistence of the infection with autoimmune features, as is the case of RF/RHD. This would explain other apparently opposite associations of ficolin-1, reported formerly in leprosy for −*542A*, −*144C*, and +*33T* (26), −*1981A* in rheumatoid arthritis (28) and earlier chronic *Pseudomonas aeruginosa* colonization in cystic fibrosis patients (27), as well as ficolin-1 deficiency in a mouse model of collagen Ab-induced arthritis (41).

Lower ficolin-1 levels were found in RF patients, an effect that might purely indicate considerable ficolin-1 consumption in RF. In contrast to our study, ficolin-1 serum levels were higher in patients with vasculitis syndrome or rheumatoid arthritis, than in those with myositis, whereas no difference was observed among patients with systemic lupus erythematosus and Behcet's disease (42). Ficolin-1 levels further correlated with several inflammatory markers, including C-reactive protein (CRP), serum amyloid protein (SAP) and complement factor C3 (42) and strongly associated with the severity of ischemic stroke, in another group (43). Interestingly, part of the benefit of intravenous immunoglobulin (IVIG) therapy relies on ficolin-1 pulldown (reported for Kawasaki disease, the most common form of acquired heart disease in childhood) (44). IVIG therapy has also been proven beneficial for Sydenham's Chorea associated with rheumatic fever (45). Moreover, anti-ficolin-1 mAb ameliorated symptoms of collagen antibody-induced arthritis (CAIA) in animal model (42). Additionally, acute injury leads to higher *FCN1* gene expression, due to specific regulatory proteins such as hypoxia factor HIF-1a (46). This collection of evidence is in line with our results associating high-*FCN1* producing promoter variants with heart damage, in later stages of the disease.

This study has some limitations, especially regarding sample size of individuals with measured ficolin-1 levels which probably affected some of the results. Increased ficolin-1 levels were observed in patients carrying the ^*^3C2 haplotype, however this effect was not evident among controls possibly due to low sample size. In addition we cannot dismiss the possibility that there may be other causal variants responsible for modulating *FCN1* expression, not investigated in this study, such as rs12377780 (in intron 1), rs7857015 (5′ upstream) and rs7858307 (3′UTR) (http://raggr.usc.edu/). Well-designed case control studies including higher number of individuals and different *FCN1* gene polymorphisms are necessary to better define the action of ficolin-1in RF. Concluding, we suggest a role for ficolin-1 in fighting GAS infection, with a possible damaging effect when infection succeeds, due to excessive complement activation. Inhibiting the final steps of complement activation may be a therapeutic clue for preventing valvar damage in patients with RHD.

## Author Contributions

FA, AB, LG, and IM-R designed the study and analyzed the data. SC performed all the assays, analyzed the data, and performed statistical tests. All of the authors contributed toward manuscript preparation and revision, and provided final approval of the version to be published.

### Conflict of Interest Statement

The authors declare that the research was conducted in the absence of any commercial or financial relationships that could be construed as a potential conflict of interest.

## Abbreviations

RF: Rheumatic Fever
RHD: Rheumatic Heart Disease
RFo: Rheumatic Fever only
*FCN1*: ficolin-1 gene
LP: Lectin Pathway
CRP: C-Reactive Protein
MAC: Membrane Attack Complex
GAS: Group A *Streptococcus*
LD: Linkage Disequilibrium
GlcNAc: N-acetyl-β-D-glucosamine
MBL: Mannose-binding lectin.
