# Supplementary material for: Sickening or Healing the Heart? The Association of Ficolin-1 and Rheumatic Fever
Source: Front Immunol. 2018 Dec 18;9:3009. doi: 10.3389/fimmu.2018.03009 (PMC6305461; doi:10.3389/fimmu.2018.03009)
Supplement: Supplementary file 1 [file Table_1.DOCX]

**TABLE S1** – Primers used for *FCN1* sequence-specific amplification

| **primer** | **5´- 3´Sequence** |
| --- | --- |
| FCN1 Prom -1981Gf | CCCATGAGCCTGGTTATC**G** |
| FCN1 Prom -1981Af | CCCATGAGCCTGGTTATC**A** |
| FCN1 Promr | ACCTCCTCTTCCTTGCAACA |
| FCN1Prom -542Gf | CAAATAATTTACTCCACC**G** |
| FCN1 Prom -542Af | CAAATAATTTACTCCACC**A** |
| FCN1 Prom -399Gf | AGATGAGGCAAGGAAGC**G** |
| FCN1Prom -399Af | AGATGAGGCAAGGAAGC**A** |
| FCN1 Ex1 +33Gr | ACTAGCAGGACAGCGAG**C** |
| FCN1 Ex1 +33Tr | ACTAGCAGGACAGCGAG**A** |
| FCN1 Prom-144 Ar | ACCAACAGGAGGTATGA**T** |
| FCN1 Prom-144 Cr | ACCAACAGGAGGTATGA**G** |
| MBL Promf* | ATGGGGCTAGGCTGCTGAG |
| MBL rev+270*  FCN2 Ex8 f*  FCN2 Ex8 r* | CCAACACGTACCTGGTTCCC  GCCAGGCCTCAGGTATAAAG  AAAGGGTTGATTGCGGAAAC |

In bold: variant nucleotides, f: forward, r: reverse, * control primers, MBL: mannose-binding lectin, Prom: promoter, Ex: exon.
